# A Hypothesis Linking Early-Life Adversity to Altered Synaptic Connectivity Through Disrupted Gut Microbiome–Microglia Communication

**DOI:** 10.3390/nu18193237

**Published:** 2026-10-01

**Authors:** Tulasi Pasam, Christian Bowers, Sahabuddin Ahmed, Arie Kaffman

**Affiliations:** Department of Psychiatry, Yale University School of Medicine, 300 George Street, Suite 901, New Haven, CT 06511, USA; tulasi.pasam@yale.edu (T.P.); cbowers357@yahoo.com (C.B.); sahabuddin.ahmed@yale.edu (S.A.)

**Keywords:** early-life adversity, microglia, gut microbiome, short-chain fatty acids (SCFAs), neuroinflammation, synaptic pruning

## Abstract

Childhood adversity is a major risk factor for the development of numerous psychiatric and medical disorders later in life. Individuals exposed to early adversity also tend to experience more severe, treatment-resistant, and highly comorbid conditions, suggesting that childhood adversity represents a distinct pathophysiological entity requiring specialized diagnostic and therapeutic approaches. However, the biological mechanisms underlying these diverse long-term outcomes remain poorly understood. Recent studies have implicated disruptions of the gut–immune–brain axis and persistent low-grade inflammation in mediating the long-term consequences of childhood adversity. Here, we propose a complementary and novel, but still speculative, hypothesis that shifts the focus away from chronic low-grade neuroinflammation and instead emphasizes the direct crosstalk between the gut microbiome and glia-mediated synaptic pruning during critical periods of brain development. Specifically, we hypothesize that alterations in microbiome-derived signals disrupt the normal maturation and function of glial cells, leading to aberrant synaptic pruning, long-lasting changes in neural circuit connectivity, and persistent deficits in cognition and emotional regulation. Although several lines of evidence are consistent with individual components of this model, direct evidence linking these processes into a causal pathway is currently limited. We therefore discuss the experimental findings that provide a rationale for this hypothesis, identify important gaps in the current literature, and propose specific experimental strategies to rigorously test its central predictions. If validated, this framework could transform our understanding of how childhood adversity becomes biologically embedded and provide a foundation for the development of novel diagnostic biomarkers and targeted therapeutic interventions.

## 1. Introduction

Early-life adversity (ELA) encompasses a diverse range of negative childhood experiences, including abuse, neglect, exposure to violence (e.g., war or neighborhood crime), environmental stressors such as pollution, and parental death or separation [1,2,3,4,5]. ELA is widely recognized as a major risk factor for abnormal brain development and is associated with increased risk for a broad range of psychiatric and medical conditions, including depression, anxiety, psychosis, substance use disorders, cardiovascular disease, arthritis, metabolic syndrome, asthma, cancer, and reduced life expectancy [1,6,7]. ELA not only elevates risk for these psychiatric disorders but is also linked to greater symptom severity and reduced responsiveness to treatment [3,8,9,10]. Moreover, individuals with a history of ELA often exhibit distinct patterns of structural and functional brain connectivity compared to those without such experiences, even when presenting with similar clinical diagnoses (e.g., depression) [11,12,13,14]. Together, these findings suggest that ELA may give rise to a distinct form of psychopathology that requires tailored intervention strategies.

**Figure 1:**
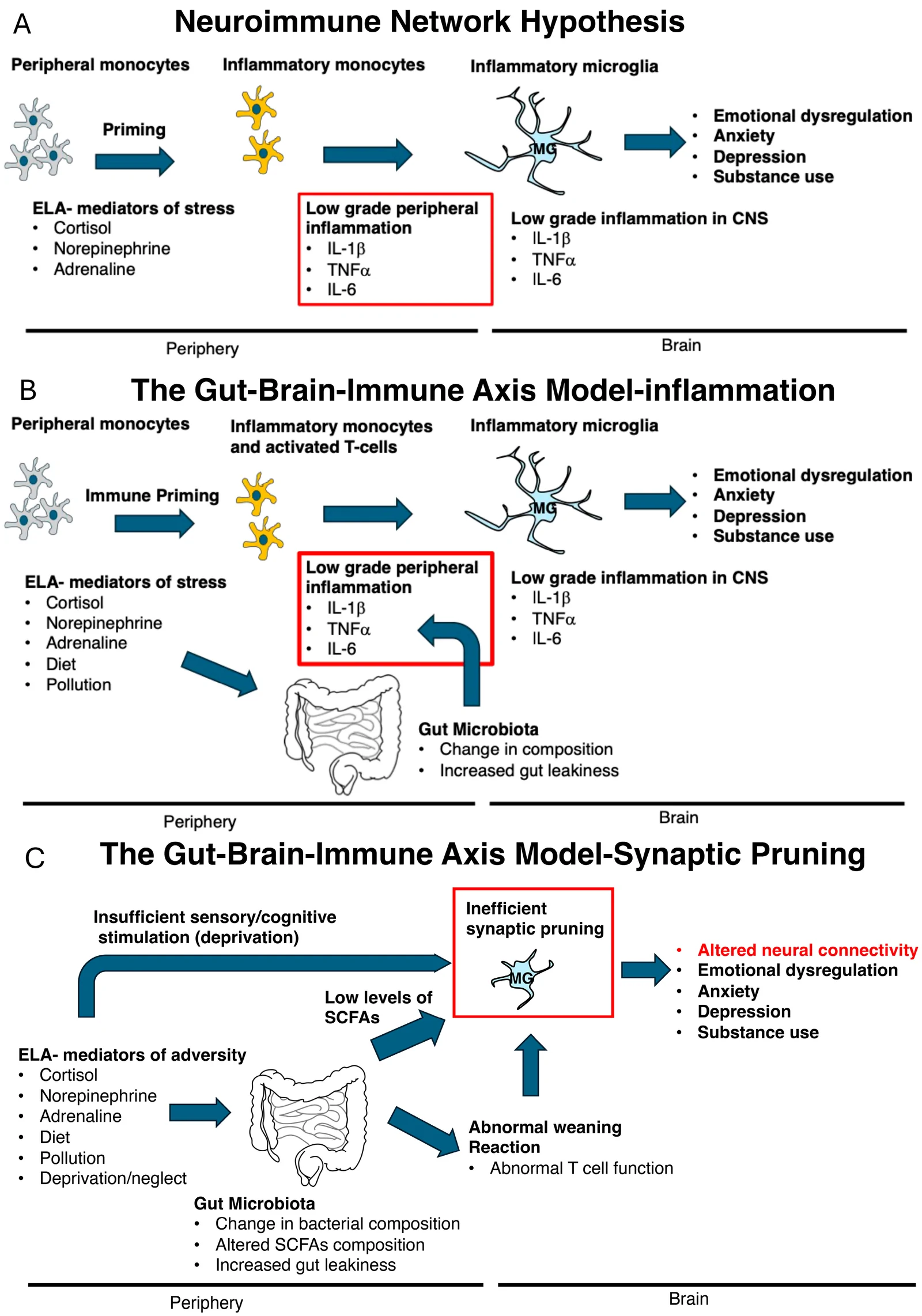
**Three Neuroimmune Models of Early-Life Adversity.** (**A**) According to the neuroimmune network hypothesis, exposure to stress early in life primes peripheral monocytes and brain microglia, leading to persistent low-grade inflammation. This chronic inflammatory state is proposed to underlie the cognitive, behavioral, emotional, and medical consequences of early-life adversity (ELA). (**B**) The gut–brain–immune–neuroinflammation model expands this hypothesis by identifying stress-induced alterations in the gut microbiome as a key upstream driver of peripheral immune activation and central neuroinflammation. (**C**) The gut–brain–immune–synaptic pruning model proposed in this review shifts the focus from chronic neuroinflammation to the role of the gut microbiome in promoting the non-inflammatory phagocytic activity of glial cells during a critical period of synaptic pruning. We propose that ELA-induced disruption of gut microbiome signaling impairs glia-mediated synaptic pruning, resulting in long-lasting alterations in neural connectivity that may, in turn, contribute to alterations in emotional regulation, anxiety, depression, substance use, cognition, and physiology. IL-1β, Interleukin-1 beta; IL-6, -Interleukin-6; MG, microglia; TNFα, Tumor necrosis factor alpha; SCFAs, Short-chain fatty acids.

Despite extensive research, the mechanisms by which ELA leads to such diverse and persistent comorbidities remain unresolved. In 2016, Nusslock and Miller proposed the neuroimmune network hypothesis, which posits that early-life stress primes peripheral innate immune cells—such as macrophages and monocytes—to produce low-grade inflammatory cytokines (e.g., IL-1β, IL-6, TNFα). These cytokines can access the brain and alter neural circuits involved in reward processing, threat detection, impulsivity, and emotional regulation. In addition, chronic low-grade inflammation has been implicated in the increased prevalence of medical conditions associated with ELA, including metabolic syndrome, arthritis, coronary artery disease, asthma, and irritable bowel syndrome [15]. This framework is supported by evidence linking ELA to modest elevations in inflammatory markers and by associations between inflammation and both psychiatric and medical outcomes [16,17,18] (Figure 1A).

More recently, this hypothesis has been expanded to incorporate the gut microbiome, giving rise to the gut–brain–immune axis model [19,20,21]. The gut microbiome, consisting of trillions of microorganisms, undergoes dynamic changes during development and plays a critical role in shaping both the immune system and brain maturation [19,20,21]. It regulates cytokine production, immune cell function, and neural development [19,20,21]. ELA has been shown to disrupt microbiome composition [19,20,21], adding another layer of complexity to its long-term effects (Figure 1B).

A key limitation of both the neuroimmune network hypothesis and current gut–brain–immune models is their emphasis on chronic low-grade inflammation as a central mechanism [15,19]. Although meta-analyses report statistically significant elevations in certain cytokines, the effect sizes are small, typically explaining only 1–4% of the variance [16,17,18]. In children and adolescents, evidence for sustained inflammation following ELA is even less consistent [18,22,23]. Similarly, rodent models of ELA generally show little to no evidence of persistent low-grade inflammation, or at best, inconsistent findings [24,25,26].

Here, we propose a modified gut–brain–immune axis hypothesis that shifts the focus away from chronic inflammation and instead highlights a more specific, though still emerging, mechanism: the interaction between ELA-induced alterations in the gut microbiome and microglia-mediated synaptic pruning during critical developmental windows. This hypothesis is grounded in a growing body of work from our laboratory demonstrating that ELA, modeled as limited bedding (LB) in mice, leads to long-term deficits in synaptic connectivity and cognition by disrupting microglia-mediated synaptic pruning during a defined developmental period [24,27,28,29]. Importantly, the gut microbiome is known to play a key role in training the immune system [30,31,32] and possibly influencing microglial development [32,33,34] during this same developmental window (Figure 1C). Although our data indicate that inflammatory cytokines are not significantly elevated in our LB model, other forms of ELA involving greater levels of physical threat may induce more pronounced inflammatory responses as illustrated in Figure 1B,C.

In this review, we first examine the role of microglia—the brain’s primary immune cells—in normal and ELA-associated neurodevelopment. We then discuss key evidence demonstrating that the gut microbiome is essential for proper immune and brain development, and we explore its potential contribution to ELA-induced alterations, with a particular focus on glia-mediated synaptic pruning. Finally, we outline critical future studies needed to test and refine this hypothesis.

## 2. The Missing Link: Microglial Mechanisms Underlying the Effects of Early-Life Adversity (ELA)

### 2.1. Microglia Play a Critical Role in Shaping Neurodevelopment

**Figure 2:**
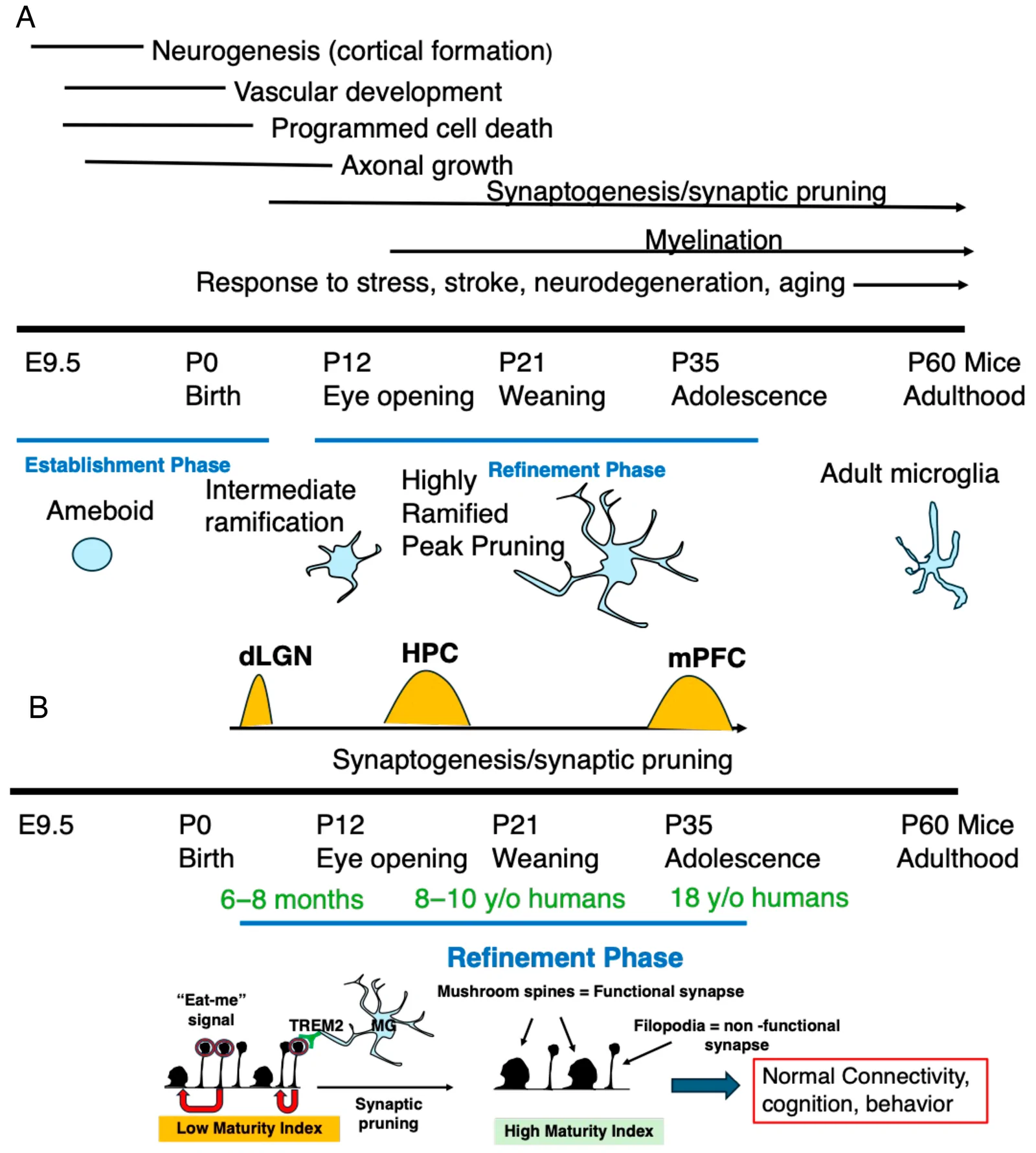
**Disruption of Microglia-Mediated Synaptic Pruning During Development Leads to Long-Term Deficits in Neural Connectivity and Behavior.** (**A**) Top. Microglia colonize the developing brain beginning around embryonic day (E) 9.5, where they play essential roles in brain development. Their functions can be broadly divided into two developmental phases: an establishment phase (~E9.5–P5), during which the basic neural architecture is formed, and a refinement phase (~P5–P40), during which neuronal circuits are sculpted and optimized. Bottom. During the establishment phase, microglia exhibit an amoeboid or sparsely ramified morphology that supports rapid proliferation, migration, secretion of trophic factors and cytokines, and phagocytic removal of apoptotic cells, excess neural progenitors, and cellular debris. During the refinement phase, microglia become highly ramified and largely sessile while continuously extending and retracting their processes to survey the surrounding tissue. These dynamic interactions support synaptogenesis, activity-dependent synaptic pruning, and myelination. (**B**) Top. During the refinement phase, synaptic pruning occurs in discrete developmental windows that vary across brain regions (orange peaks). Bottom. Before pruning, immature neural circuits contain a high density of thin, immature dendritic spines and relatively few mature mushroom spines, resulting in a low synaptic maturity index (the ratio of mature to immature spines). During peak pruning, weak or inactive synapses are labeled by “eat-me” signals (red circles), which are recognized by phagocytic receptors such as TREM2 (green) expressed on microglia (MG). Selective elimination of these non-functional synapses reallocates structural and metabolic resources within the dendrite (red arrow) to strengthen and stabilize functional synapses, leading to an increased synaptic maturity index, improved circuit connectivity, and normal cognitive and behavioral development. Conversely, disruption of microglia-mediated synaptic pruning during these critical developmental windows results in persistent abnormalities in neural connectivity, cognition, and behavior. The approximate equivalent timeline in humans is shown in green. Dorsal lateral geniculate nucleus (dLGN), Hippocampus (HPC), Medial prefrontal cortex (mPFC).

Microglia are the tissue-resident macrophages of the central nervous system that migrate into the brain beginning at embryonic day (E) 9.5, where they play an instrumental role in regulating and coordinating multiple developmental processes [26,35,36]. Conceptually, these processes can be divided into two distinct phases: an establishment phase (~embryonic day 9.5 (E9.5)–postnatal day 5 (P5)), during which the basic neural architecture is formed, and a refinement phase (~P5–P40 in mice and ~6 months–18 years in humans), during which neuronal circuits are sculpted and optimized (Figure 2). During the establishment phase, microglia are highly motile amoeboid or sparsely ramified cells that proliferate and disperse throughout the brain parenchyma, eventually accounting for approximately 10% of all brain cells. Early in this phase, microglia orchestrate cortical development by regulating neural stem cell proliferation, migration, differentiation, and survival [35,36]. These functions are mediated through the expression of chemokine receptors (e.g., CX3CR1, CX3CR4), purinergic receptors (e.g., P2RY12), and phagocytic receptors (e.g., MerTK and TREM2), which guide their migration and facilitate the clearance of apoptotic cells and cellular debris. Microglia also secrete cytokines (e.g., IL-1β and TNFα), growth factors (e.g., NGF and IGF1), and chemokines (e.g., CCL2 and CCL5) that regulate neurogenesis, vascular development, axonal growth, and synapse formation [26,35,36].

By the end of the establishment phase, the developing brain has formed an initial synaptic network characterized by an excess of synaptic connections, many of which are weak, non-functional, or inappropriate (Figure 2A,B). During the refinement phase, microglia become highly ramified and largely sessile while continuously extending and retracting highly dynamic cellular processes decorated with numerous sensory and phagocytic receptors at remarkable speeds (0.5–7.6 μm/min) [37,38,39,40,41]. This constant surveillance enables the efficient removal of excess and non-functional synapses through a process known as activity-dependent synaptic pruning. Synaptic pruning requires neuronal activity to distinguish functional from non-functional synapses and label the latter with “eat-me” signals. These signals are recognized by phagocytic receptors expressed on microglia, including TREM2, allowing selective elimination of redundant or inappropriate synapses [42,43]. The removal of weak synapses promotes the maturation and stabilization of stronger, functional synaptic connections—potentially by reallocating structural and metabolic resources within the dendrite—resulting in more precise and efficient neuronal communication that is essential for accurate sensory processing, learning, and memory later in life [24,44,45] (Figure 2B).

Different brain regions undergo peak synaptic pruning at distinct developmental ages that closely correspond to periods of peak innervation, neuronal activity, and functional maturation [42,43]. For example, synaptic pruning in mice peaks in the dorsal lateral geniculate nucleus (dLGN) between P5–P8 [44], in the developing hippocampus during the juvenile period (approximately P14–P18) [28,46,47], and in the prefrontal cortex during adolescence (approximately P40–P45) [48] (Figure 2B). These transient waves of enhanced pruning are characterized by increased expression of “eat-me” signals on weak synapses together with a marked increase in microglial size and phagocytic capacity [28,43,47]. The requirement for neuronal activity to initiate synaptic pruning is particularly relevant for understanding the mechanisms underlying the long-term consequences of chronic neglect and deprivation (see Section 2.3).

Following these developmental windows, rates of synaptic pruning decline approximately 5- to 8-fold and stabilize at the relatively low levels observed in adulthood [24,28,43,47]. As described below, disruption of synaptic pruning during these critical developmental periods leads to persistent abnormalities in neural connectivity, cognition, and behavior (Figure 2B).

In the mature brain, microglia continue to regulate homeostatic synapse formation and elimination, although these processes occur at substantially lower rates than during development [24,40,49]. Nevertheless, microglia remain critical mediators of the brain’s response to major insults, including psychological trauma, traumatic brain injury, stroke, and neurodegenerative disease [24] (Figure 2A).

### 2.2. The Rationale for Studying Microglia as Key Mediators of Neurodevelopmental Consequences Following Early-Life Adversity

Several lines of evidence suggest that microglia play a central role in mediating the effects of ELA. As discussed above, microglia regulate multiple developmental processes throughout brain maturation (Figure 2), and their activity is highly sensitive to environmental changes associated with ELA, including stress, sensory input, neuronal activity, and nutritional state [24,26]. Moreover, microglial activity peaks during relatively narrow developmental windows, and perturbations during these critical periods produce long-lasting alterations in neural connectivity, behavior, and physiology [24,26]. For example, transient elimination of microglia in the prefrontal cortex (PFC) during adolescence, when synaptic pruning peaks in this region, causes abnormal synaptic connectivity and impaired PFC function in adulthood—effects that are not observed when microglia are eliminated in adulthood [48]. Similarly, transient depletion of microglia during peak synaptic pruning in the hippocampus (P13–21) results in persistent deficits in synaptic connectivity and hippocampal function [28]. Deficient microglia-mediated pruning in the developing dorsal lateral geniculate nucleus (dLGN) leads to long-term impairments in the segregation of retinal inputs from the left and right eyes and the abnormal retention of weak retinal ganglion cell synapses [44]. Likewise, abnormal microglial activity during early development disrupts normal sexual [50] and social behaviors later in life [45,51]. In the developing visual cortex, microglia eliminate non-functional synapses during a precisely defined critical period in response to monocular deprivation [52]. This process results in a permanent loss of vision from the deprived eye that cannot be reversed by restoring visual input later in life and is not observed when monocular deprivation occurs in adulthood [52,53].

Additional evidence comes from studies demonstrating that ELA disrupts microglial development and function in rodents and non-human primates [24,54,55]. Although many of these studies are correlative, several recent reports provide causal evidence linking impaired microglia-mediated synaptic pruning during the postnatal period to long-term deficits in neural connectivity and behavior in mice exposed to ELA using the limited bedding (LB) model [27,28,29]. LB impairs the ability of microglia to phagocytose non-functional synapses during peak pruning with relatively little evidence of a classical pro-inflammatory response [27,28,29]. An emerging finding across these studies is the presence of marked sex differences in the response to ELA [28] (see Section 2.3).

Evidence also indicates that ELA alters microglial responses to “secondary hits” encountered later in life [24]. Compared with control animals, rodents exposed to ELA exhibit exaggerated microglial activation following subsequent immune or neurological insults, leading to more severe behavioral and neurological outcomes [24]. For example, daily exposure to an adult CD1 male between P14 and P21 exacerbates neurological deficits following traumatic brain injury (TBI) in adulthood compared with mice that were not exposed to ELA. This increased vulnerability is associated with enhanced microglial secretion of IL-1β in response to injury [56].

Taken together, these findings suggest that microglia are uniquely positioned to mediate the long-term neurodevelopmental consequences of ELA. They regulate multiple developmental processes that are highly sensitive to environmental input during discrete critical periods, and disruption of their activity during these windows alters synaptic connectivity, circuit maturation, and behavior later in life. Accumulating evidence from rodent and non-human primate studies further demonstrates that ELA disrupts microglial development and function, including microglia-mediated synaptic pruning, while sensitizing microglia to secondary insults encountered in adulthood. Together, these findings support the hypothesis that abnormal microglial signaling and remodeling during development represent a key mechanism linking ELA to persistent alterations in brain function and increased vulnerability to psychopathology later in life.

### 2.3. Disrupted Microglial Synaptic Pruning During a Critical Developmental Window Produces Sex-Specific Impairments in Synaptic Connectivity and Cognition in a Mouse Model of Childhood Neglect

ELA encompasses a wide range of negative childhood experiences, including childhood hardship, abuse, neglect, extreme poverty, and exposure to violence [3,57,58]. In 2023, the U.S. Department of Health and Human Services reported 546,159 confirmed cases of child maltreatment, approximately three-quarters of which involved neglect. Childhood neglect is broadly defined as conditions in which a child’s basic needs are not adequately met, resulting in disruption of normal development [59]. This definition encompasses both parental omissions and environmental impoverishment [59] and is associated with a unique and severe set of cognitive, social, and structural abnormalities not commonly observed in other forms of ELA, such as physical or sexual abuse. These abnormalities include stunted growth, avoidant attachment, cortical thinning, cognitive deficits, hyperactivity, and abnormal myelination [59,60]. Despite its prevalence, childhood neglect remains one of the least studied forms of ELA [59,60,61], and no animal models have faithfully reproduced key features of childhood deprivation [27,62,63].

To address this gap, we employed a modified limited bedding and nesting (LB) paradigm in which pups are raised under impoverished conditions consisting of a 75% reduction in corncob bedding and the absence of nesting material (i.e., environmental impoverishment). The absence of bedding and nesting material induces an erratic pattern of maternal care (i.e., parental omission), leading to many of the key clinical features observed in neglected children, including avoidant attachment, cortical thinning, hyperactivity, stunted growth, and deficits in hippocampal-dependent memory [24,27,63,64]. Furthermore, providing LB pups with toys in the home cage from P14–25 normalizes hippocampal-dependent deficits observed later in life, further supporting the notion that LB represents a mouse model of childhood neglect [27].

**Figure 3:**
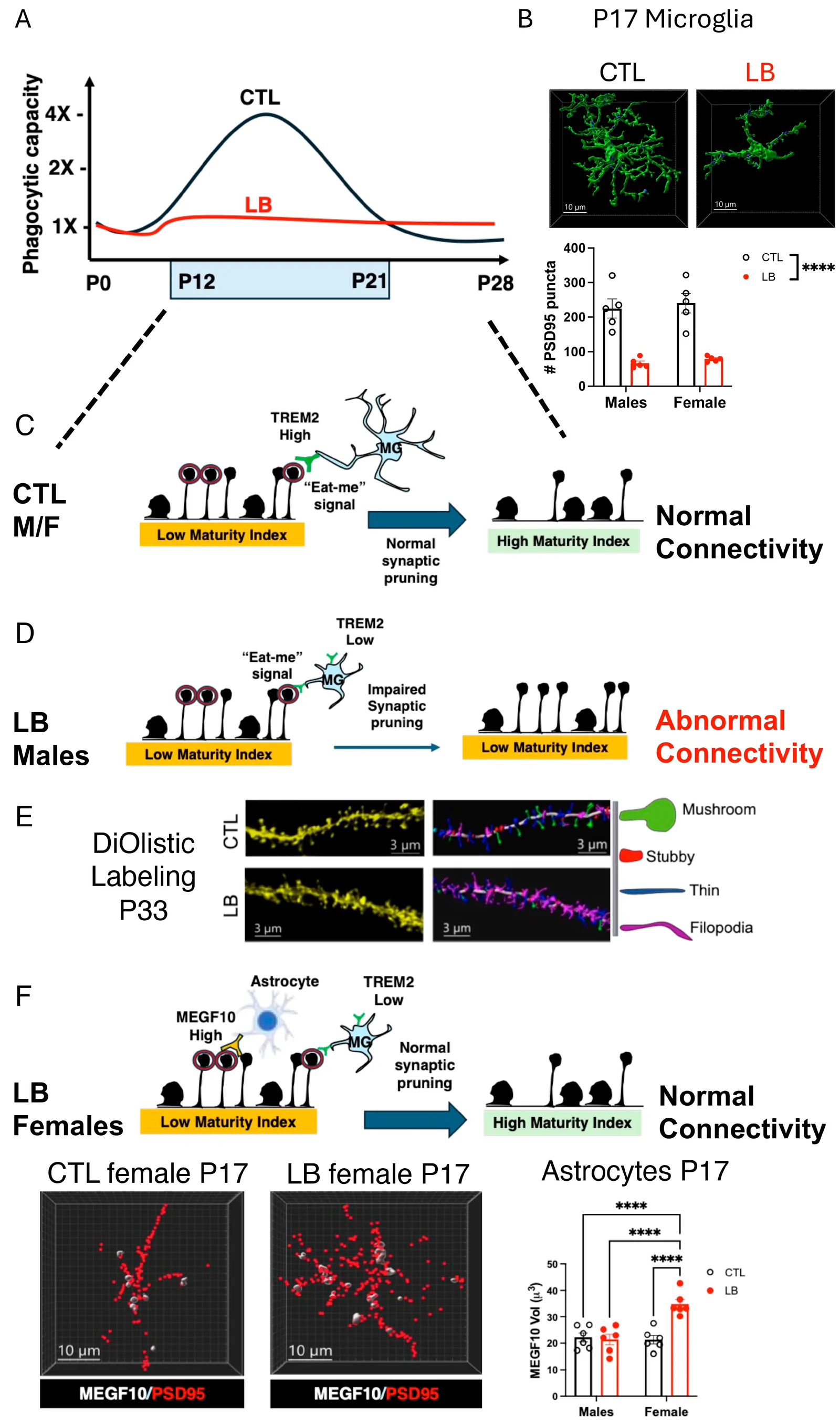
**Disruption of Microglia-Mediated Synaptic Pruning During a Critical Developmental Window Causes Sex-Specific Impairments in Synaptic Connectivity and Cognition in Adolescent LB Mice**. (**A**) Microglia-mediated synaptic pruning in the hippocampus peaks during the second and third weeks of life in control (CTL) mice but is markedly reduced in LB mice. (**B**) Top: Representative images of microglia from P17 CTL and LB mice. Bottom: LB reduces the number of PSD95 puncta engulfed by microglia at P17 in both males and females [27,28,29]. (**C**) During peak pruning, microglia in CTL mice increase in size, exhibit enhanced phagocytic activity, and express high levels of TREM2 [27,29]. This ensures efficient removal of non-functional synapses and promotes normal synaptic connectivity and hippocampal function later in life [27,28,29]. (**D**) Microglia from LB male mice fail to effectively prune synapses during this critical developmental period, in part because of reduced expression of the TREM2 receptor, leading to impaired synaptic connectivity and abnormal hippocampal function later in life. (**E**) DiOlistic images and corresponding Imaris models of apical dendrites in the stratum radiatum of adolescent CTL and LB male mice [28]. Mature mushroom spines are shown in green, and immature spines are shown as thin (blue) and filopodia (magenta). Note the increased abundance of immature spines and reduced abundance of mature mushroom spines in LB mice. (**F**) Top: LB female mice exhibit deficits in microglial function similar to those observed in their male littermates (see Figure 3B). However, astrocytes increase synaptic pruning, providing a potential compensatory mechanism that allows females to maintain normal synaptic connectivity and hippocampal function despite persistent deficits in microglial function. Bottom: LB females show increase PSD95 engulfment and MEGF10 expression in astrocytes [28]. MEGF10-Multiple EGF-like domains protein 10, MG- Microglia, TREM2 -Triggering receptor expressed on myeloid cells 2. **** *p* < 0.0001.

Although many of these abnormalities occur in both sexes [63,64], deficits in hippocampal function are significantly more pronounced in males [27,28]. To better understand these deficits, we used DiOlistic labeling to examine the effects of rearing and sex on spine density and morphology in CA1 pyramidal neurons in adolescent (P33) mice. We found that LB males, but not females, exhibited a reduced synaptic maturity index, defined as the ratio of mature mushroom spines to immature spines (Figure 3). Furthermore, the synaptic maturity index was significantly correlated with contextual freezing behavior, such that mice with a higher synaptic maturity index exhibited enhanced contextual freezing [28]. Using immunohistochemistry and high-resolution confocal microscopy, we confirmed reduced glutamatergic synapse density in the stratum radiatum of CA1 pyramidal neurons in LB males, but not female littermates [28]. Similar sex-specific deficits were observed using resting-state functional MRI (rsfMRI), which revealed reduced local functional connectivity in the dorsal hippocampus of LB males but not LB females [28]. Importantly, rsfMRI also identified reduced local functional connectivity in several additional brain regions in LB males but not LB females, including the entorhinal cortex, striatum, basolateral amygdala, hypothalamus, and nucleus accumbens, indicating that these sex-specific deficits are not restricted to the hippocampus. These findings suggest that impaired microglia-mediated synaptic pruning may disrupt the maturation of distributed neural circuits. Moreover, because rsfMRI can be readily applied in humans, this approach provides a promising translational tool to determine whether similar alterations in functional connectivity are present in children and adolescents exposed to severe neglect [28].

The presence of a large number of immature dendritic spines in the LB hippocampus suggested deficits in microglia-mediated pruning [28,29] and prompted us to examine the effects of LB on microglial phagocytic activity at P17, when synaptic pruning peaks in the hippocampus [28,46,47,65]. We found significant deficits in microglia-mediated synaptic pruning, evidenced by reduced microglial size, ramification, phagosome size, and engulfment of synaptic material (PSD95) [28,29] (Figure 3A). Phagocytic deficits were also observed using an ex vivo phagocytic assay in which purified microglia were assessed for their ability to phagocytose pHrodo-labeled synaptosomes [29]. Importantly, these pruning deficits were observed in both males and females [27,28,29] and were no longer present at P33, when pruning activity naturally declines [28].

These findings led us to hypothesize that LB disrupts microglial phagocytic capacity during the second and third weeks of life, when pruning activity peaks in the hippocampus. Deficits during this critical developmental period lead to the retention of large numbers of immature spines, resulting in abnormal synaptic maturation and impaired contextual fear conditioning, effects that are, for unclear reasons, more pronounced in males (Figure 3).

To test this hypothesis, we used transgenic mice that allow transient ablation of microglia during the second and third weeks of life. Remarkably, transient microglial depletion reproduced the same synaptic and behavioral abnormalities observed in LB mice, including reduced synaptic maturity, decreased glutamatergic synapse density, impaired hippocampal connectivity, and deficits in contextual fear conditioning (CFC), all of which were male-specific [28]. We next asked whether enhancing microglial activity during this developmental window could reverse the effects of LB. Activation of microglia during this period normalized microglial phagocytic activity at P17, restored synaptic maturation, and rescued deficits in hippocampal connectivity and CFC in adolescent LB males [28].

The observation that transient microglial depletion or exposure to LB did not produce the same synaptic and hippocampal deficits in females suggested that females may compensate for deficits in microglia-mediated synaptic pruning. Since astrocytes also participate in synaptic pruning and can compensate for deficits in microglial pruning [66,67,68,69,70], we examined the effects of LB on astrocytic pruning in male and female mice. Indeed, LB females exhibited enhanced astrocytic synaptic pruning activity that was not observed in LB males. This increase in astrocytic pruning was accompanied by elevated expression of the phagocytic receptor MEGF10 [28]. Because MEGF10 is required for astrocyte-mediated synapse elimination [66], these findings suggest that LB females compensate for impaired microglial pruning by upregulating MEGF10 expression and phagocytic activity in astrocytes [28] (Figure 3F).

More recently, we showed that LB impairs microglial phagocytic activity, at least in part, by reducing expression of the receptor TREM2 [27]. TREM2 is selectively expressed by microglia and is required for recognizing “eat me” signals decorating non-functional synapses [71,72,73,74,75] (Figure 3A,B). LB significantly reduced microglial TREM2 expression across multiple mouse strains [27] (Figure 3C). Additional studies using knockout mice revealed that LB impairs synaptic pruning through both TREM2-dependent and TREM2-independent mechanisms [27]. However, overexpression of TREM2 in microglia was sufficient to normalize phagocytic activity at P17, restore synaptic maturity and glutamatergic synapse density, and rescue deficits in hippocampal connectivity and contextual fear conditioning in adolescent LB mice [27]. Genomic studies further confirmed that LB impairs the ability of microglia to phagocytose non-functional synapses during peak pruning with relatively little evidence for a classical pro-inflammatory response [29].

Because the LB paradigm models childhood neglect [27,28,29,63,64], we next examined whether early-life enrichment could restore microglial function. Brief environmental enrichment during the peak pruning window normalized TREM2 expression, restored microglial phagocytic activity, and rescued contextual fear conditioning deficits in adolescent LB males [27]. Importantly, these restorative effects were absent in Trem2 knockout mice, demonstrating that TREM2 is required for enrichment-induced restoration of microglial function [27]. Together, these findings reveal a critical role for microglial TREM2 signaling in mediating the effects of deprivation and enrichment on synaptic pruning and hippocampal development [27].

In summary, LB impairs the ability of microglia to recognize and phagocytose non-functional synapses during a critical developmental period when the majority of pruning occurs in the hippocampus, with relatively little evidence for a significant pro-inflammatory response. Abnormal pruning leads to the retention of immature spines that interfere with the formation of mature and efficient synaptic connectivity necessary for normal cognition. This developmental process is partly regulated by cognitive deprivation and stimulation within the home cage (Figure 1C) and, as proposed in the next section, is also likely influenced by the gut microbiome, possibly with important implications for the upregulation of astrocytic pruning in females.

## 3. Central Hypothesis: Early-Life Adversity Disrupts the Developing Gut Microbiome, Leading to Impaired Glia-Mediated Synaptic Pruning During a Critical Period of Brain Development

### 3.1. The Hypothesis

The gastrointestinal tract of adult mammal harbors approximately 10^12^ microorganisms, collectively referred to as the gut microbiota [19,21,31,76]. The gut microbiome, which encompasses the collective genomes of these microorganisms, contains over 100-fold more genes than the mammalian genome and functions as a metabolic and immunological extension of the host [31,76]. In addition to protecting against pathogens and metabolizing dietary components and drugs, the gut microbiota produces a diverse array of bioactive molecules, including short-chain fatty acids (SCFAs), vitamins, bile acids, and neurotransmitters, that profoundly influence host physiology [19,21,31,76,77,78].

Beyond its metabolic functions, the gut microbiota continuously exposes the host to microbial-associated molecular patterns and other immunogenic molecules that educate and shape the developing immune system [30,31,32]. These interactions promote immune homeostasis and protect against inflammatory disorders, but dysbiosis can also contribute to chronic inflammation and increase susceptibility to diseases such as inflammatory bowel disease, coronary artery disease, diabetes, and neurodegenerative disorders [30,76,79]. As discussed below, microbial metabolites and immune signaling also play critical roles in regulating normal brain development, stress reactivity, and neuroimmune function. Conversely, the host immune system shapes the composition of the gut microbiota, creating a dynamic bidirectional feedback loop that maintains physiological homeostasis [19,76,79].

**Figure 4:**
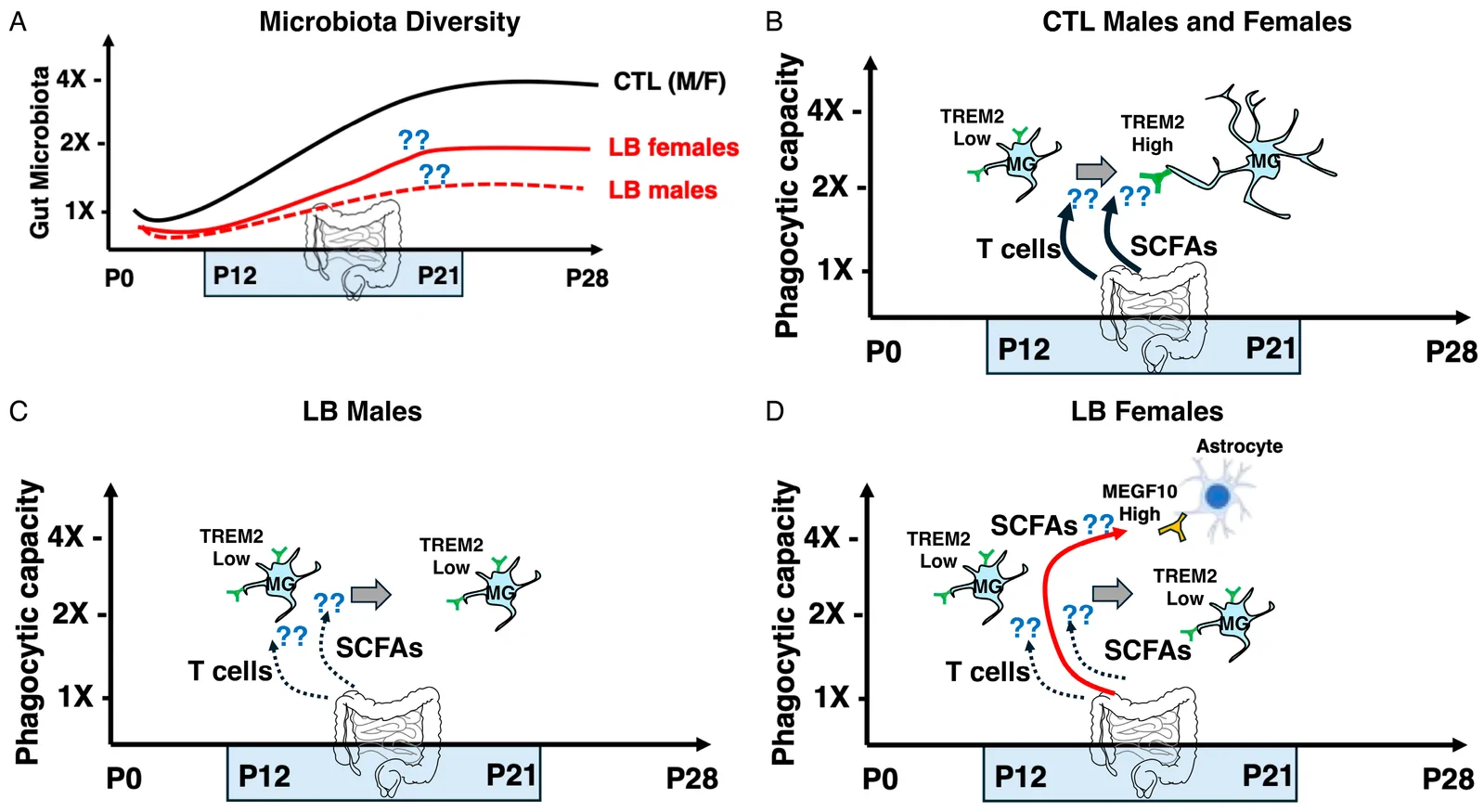
**Proposed Hypothetical Model Linking Early-Life Adversity, the Gut Microbiome, and Glia-Mediated Synaptic Pruning During Hippocampal Development.** The model summarizes our current hypothesis, with major mechanistic relationships that remain to be established experimentally indicated by question marks (??). (**A**) Limited bedding (LB) induces sex-specific alterations in the gut microbiome during the peak period of synaptic pruning in the developing hippocampus. (**B**) In control (CTL) mice, signals derived from the gut microbiome, including short-chain fatty acids (SCFAs) and functional T cells, promote the developmental increase in TREM2 expression and phagocytic activity required for efficient microglia-mediated synaptic pruning. Microglia (MG). Although SCFAs have been shown to influence microglial function in adulthood, their role in regulating microglial function during this critical developmental period, particularly in LB mice, remains unclear. Similarly, although impaired T-cell entry into the brain during this critical period produces deficits in microglial function similar to those observed in LB mice, it remains unknown whether LB disrupts T-cell entry into the developing brain. (**C**) In LB male and female mice, alterations in the gut microbiome reduce the production of signals required to support efficient microglia-mediated synaptic pruning, resulting in impaired synaptic refinement. (**D**) Sex-specific changes in the gut microbiome of LB female mice promote increased astrocyte-mediated synaptic pruning (red arrow), providing a potential compensatory mechanism that preserves synaptic connectivity despite persistent deficits in microglial function. Females- F, Males- M, MEGF10-Multiple EGF-like domains protein 10, MG- Microglia, TREM2 -Triggering receptor expressed on myeloid cells 2, SCFAs-Short-chain fatty acids.

Here, we propose that LB-induced alterations in gut microbiota composition during the second and third postnatal weeks impair microglia-mediated synaptic pruning during this critical developmental window, resulting in persistent abnormalities in neural connectivity and cognition. Furthermore, we hypothesize that sex-specific changes in the gut microbiome promote a compensatory increase in astrocyte-mediated synaptic pruning in LB females (Figure 4).

Although speculative, this hypothesis is supported by several converging observations. First, the gut microbiota undergoes dramatic remodeling during the second and third weeks of life as offspring transition from maternal milk to solid food [30]. This developmental transition triggers a transient peripheral immune response known as the weaning reaction, which is essential for establishing a functional population of T cells and promoting lifelong immune tolerance [30]. The weaning reaction is driven, in part, by increased production of SCFAs by the developing gut microbiota and displays many hallmarks of a critical period, as it must occur within a defined developmental window and cannot be fully recapitulated in adulthood [30].

If microbiota-derived SCFAs are required to educate T cells during this period, it is plausible that they also regulate microglia-mediated synaptic pruning, which peaks during the same developmental window. Consistent with this hypothesis, a series of elegant studies from the Prinz laboratory demonstrated that SCFAs, particularly acetate, are essential for maintaining adult microglial homeostasis [80,81]. In germ-free (GF) mice, the absence of a gut microbiota alters microglial morphology, transcriptional profile, mitochondrial metabolism, and immune function [80,81]. Although these microglia have often been described as “immature [80,81],” their phenotype is perhaps better characterized as metabolically compromised [81]. Microglia from GF mice exhibit increased mitochondrial number but reduced mitochondrial efficiency due to impaired oxidative phosphorylation, likely resulting from reduced activity of mitochondrial complex II [81]. Consequently, these cells are less capable of mounting effective immune responses to lipopolysaccharide (LPS) or viral infection [80].

Importantly, depletion of the gut microbiota markedly reduces acetate levels in the brain, while acetate supplementation restores many of the morphological, transcriptional, and mitochondrial abnormalities observed in GF microglia [80,81]. Whether acetate also rescues impaired inflammatory responses to LPS or viral infection remains unknown, as do the precise mechanisms by which acetate regulates mitochondrial metabolism and how these changes influence process motility and synaptic phagocytosis. Nevertheless, these studies establish a compelling and direct mechanistic link between the gut microbiome and microglial function.

The ability of acetate supplementation to rescue many microglial abnormalities in adult GF mice suggests that the gut microbiota continuously regulates microglial physiology throughout life [80,81]. A similar dynamic relationship has been described for formation of the blood–brain barrier (BBB). SCFAs produced by the gut microbiota promote expression of endothelial tight junction proteins and are required for normal BBB integrity. BBB defects observed in GF mice can be reversed in adulthood by either microbial colonization or administration of sodium butyrate [82].

In contrast, several other microbiota-dependent processes appear to occur only during restricted developmental windows. As discussed above, the weaning reaction represents a critical period for the establishment of T-cell function and long-term immune homeostasis [30]. Likewise, germ-free mice exhibit exaggerated hypothalamic–pituitary–adrenal (HPA) axis responses to stress in adulthood, and this phenotype can be rescued only by microbial colonization early in life, but not during adulthood [83]. Collectively, these observations suggest that the gut microbiota regulates brain and immune function through both lifelong homeostatic mechanisms and discrete developmental critical periods. We propose that microglia-mediated synaptic pruning during the second and third postnatal weeks represents one such critical period during which microbiota-derived signals are required for normal brain maturation (Figure 4).

### 3.2. Gaps and Future Directions

Numerous studies have demonstrated that ELA alters gut microbiota composition across multiple mammalian species, including rodents [84,85,86,87], nonhuman primates [88,89,90], and humans [19,91]. However, most rodent studies have relied on the maternal separation model [85,86,87], with only a single study directly examining the effects of LB on the gut microbiome [84]. This study is particularly relevant because it demonstrated significant alterations in gut microbiome composition in LB mice at weaning, a developmental period characterized by intense synaptic pruning in the hippocampus (Figure 2). Nevertheless, as discussed below, these findings require replication, and additional studies are needed to determine whether LB-induced alterations in the gut microbiome are causally linked to changes in synaptic pruning.

Most maternal separation studies have focused on the therapeutic potential of probiotics, defined as live microorganisms that confer a health benefit when administered in adequate amounts, to ameliorate behavioral abnormalities associated with ELA (reviewed in [85]). These studies provide compelling preclinical evidence that alterations in the gut microbiota contribute to behavioral deficits induced by ELA. However, several important questions remain unresolved. First, few studies have determined whether probiotic treatment normalizes gut microbiota composition or restores key microbial metabolites such as SCFAs. Second, probiotic administration has generally been initiated during adulthood [86,87], despite evidence that some microbiota-dependent developmental processes are most sensitive during early postnatal life. Finally, the cellular and molecular mechanisms by which probiotics improve behavioral outcomes remain poorly understood.

Addressing these gaps will require a more comprehensive characterization of microbiota development during the second and third postnatal weeks, when the gut microbiome undergoes rapid remodeling in parallel with peak levels of glia-mediated synaptic pruning (Figure 4A). In addition to characterizing microbiota composition, future studies should quantify SCFA concentrations in both the gut and brain, as brain levels are likely to be more directly relevant to the regulation of synaptic pruning. Because ELA produces marked sex differences in both behavior and glial function—including the compensatory increase in astrocyte-mediated pruning that we observe in LB females [28]—these studies should include both sexes and focus on the critical developmental period encompassing the second and third postnatal weeks (Figure 4).

Although identifying alterations in microbiota composition and SCFA production is essential, establishing causality represents the next major challenge. One approach would be to colonize germ-free (GF) pups raised under control conditions with fecal microbiota obtained from P17 control or LB offspring. Colonization should occur at approximately P12, before the peak of hippocampal synaptic pruning, followed by assessment of microglial phagocytic activity, transcriptional and epigenetic profiles, and synaptic engulfment at P17. We predict that transfer of microbiota from LB donors will recapitulate not only the microglial phenotype observed at P17 but also the abnormalities in synaptic connectivity and cognition that emerge later in life (Figure 3). It would be particularly informative to determine whether microbiota derived from LB females selectively enhances astrocyte-mediated phagocytosis, consistent with the sex-specific compensatory mechanisms described above (Figure 3F and Figure 4D). Such studies should include appropriate control groups, including vehicle-treated GF, CTL, and LB mice, to account for known abnormalities in microglial morphology [80,81] and BBB permeability in GF mice [82]. Sufficient numbers of independent litters should be included in the analysis, and the potential effects of these manipulations on maternal care should also be evaluated.

Successful completion of these studies would provide direct evidence that ELA-induced alterations in the gut microbiota causally disrupt developmental synaptic pruning. Because maternal separation and LB produce distinct effects on microglial pruning [24], comparing microbiota transfer from these two models may also identify shared versus paradigm-specific microbial mechanisms. The translational relevance of these findings could subsequently be explored by colonizing GF mice with fecal samples obtained from well-characterized cohorts of children exposed to different forms of ELA [91]. Parallel clinical studies should determine whether ELA alters fecal and circulating SCFA concentrations and whether these changes correlate with hippocampal function and local functional connectivity, which may serve as non-invasive biomarkers of abnormal synaptic pruning [27,28].

If LB is found to reduce SCFA levels, particularly within the brain, an important next step will be to determine whether supplementation with physiologically relevant mixtures of SCFAs during the second and third postnatal weeks rescues deficits in microglia- and astrocyte-mediated synaptic pruning observed in LB mice. Existing studies have almost exclusively examined the effects of SCFAs on adult microglia [33,34,80,81], and it remains unknown whether microglial pruning is impaired during the peak developmental pruning period in GF mice or whether these deficits can be rescued by SCFA supplementation. Importantly, SCFA supplementation in conventionally colonized LB mice would also help circumvent some of the developmental abnormalities inherent to GF models that can complicate interpretation of experimental outcomes (see above). Extending these studies to nonhuman primates and humans will also be essential. Maternal separation has recently been shown to impair microglia-mediated synaptic pruning in the developing amygdala of nonhuman primates [54,55], providing an opportunity to determine whether these abnormalities are associated with alterations in SCFA levels and whether they can be corrected by SCFA supplementation. Similarly, recent advances in human brain organoid models containing microglia now permit mechanistic studies of human microglial development [92,93,94]. Because organoid-derived microglia often fail to acquire a fully homeostatic transcriptional profile [92,93,94], it will be important to determine whether SCFAs promote their maturation.

Although the molecular mechanisms by which microbiota-derived SCFAs—particularly acetate—support mitochondrial complex II activity remain incompletely understood, this pathway currently represents the most direct and best-supported mechanism linking the gut microbiota to microglial function. Efficient mitochondrial ATP production is likely to be especially important during the postnatal period, when microglia exhibit highly dynamic process motility and exceptionally high rates of endocytosis and synaptic phagocytosis (Figure 1C and Figure 4). Nevertheless, additional, non-mutually exclusive mechanisms should also be considered. One possibility is that reductions in SCFA production impair blood–brain barrier (BBB) maturation. SCFAs promote expression of endothelial tight junction proteins [82], and ELA-induced reductions in SCFA levels may increase BBB permeability, exposing the developing brain to circulating factors that disrupt microglial function and synaptic pruning (Figure 4). A second possibility involves adaptive immunity. Recent studies demonstrate that microbiota-dependent immune maturation during the second and third postnatal weeks promotes activation and differentiation of CD4^+^ T cells, which subsequently enter the CNS and contribute to normal microglial maturation [32]. Consistent with this model, depletion of CD4^+^ T cells during early postnatal life, but not after weaning, impairs microglial maturation, reduces synaptic pruning, and produces long-term behavioral abnormalities, including deficits in contextual fear conditioning [32]. Together, these findings suggest that ELA-induced disruption of the developing gut microbiome may impair glia-mediated synaptic pruning through multiple convergent mechanisms, including altered SCFA-dependent mitochondrial metabolism, impaired BBB maturation, and defective microbiota-dependent activation of CD4^+^ T cells (Figure 4).

Finally, although our focus here is on the potential role of gut-derived signals in regulating synaptic pruning during this critical developmental period, other factors should also be considered and may interact with gut-derived signals to influence this process. These include reduced neuronal activity due to the impoverished LB environment [27], increased neurogenesis observed in LB mice at this age [95,96], changes in stress-related mediators, and hormonal regulation [26]. One approach we are currently pursuing involves the use of proteomics, genomics, and spatial transcriptomics to identify, in an unbiased manner, the molecular pathways through which LB alters microglial function during this critical developmental period.

## 4. Conclusions

Recent work from our laboratory and others has established that abnormal microglial activity during early postnatal life is a direct cause of persistent deficits in neural connectivity and behavior in rodent models of ELA [27,28,97]. Similar abnormalities in microglia-mediated synaptic pruning have recently been reported in nonhuman primates exposed to ELA [54,55], suggesting that this mechanism is evolutionarily conserved and may also contribute to neurodevelopmental disorders in humans. Our studies further demonstrate that LB impairs developmental synaptic pruning by reducing expression of the microglial receptor TREM2, although additional upstream mechanisms are likely to contribute [27].

In this review, we hypothesize that disruption of the developing gut microbiome represents one such mechanism. We summarize evidence supporting a model in which ELA-induced alterations in gut microbial composition and metabolism impair glia-mediated synaptic pruning through multiple, potentially convergent pathways, including reduced production of SCFAs, altered mitochondrial function, impaired blood–brain barrier maturation, and defective microbiota-dependent activation of CD4^+^ T cells. Although many aspects of this speculative model remain to be tested and validated, it provides a coherent mechanistic framework linking environmental adversity, the developing immune system, and abnormal circuit formation.

We also identify key experimental approaches needed to establish causality, including longitudinal characterization of microbiota development, fecal microbiota transplantation, SCFA supplementation during defined developmental windows, and mechanistic studies in germ-free mice, nonhuman primates, and human model systems. These experiments will determine whether alterations in the gut microbiome are merely associated with, or are directly responsible for, impaired developmental synaptic pruning. If supported, this hypothesis would fundamentally expand our understanding of how early-life experiences shape brain development and could identify the gut microbiome as a modifiable therapeutic target. Interventions aimed at restoring microbiota composition or microbial metabolites, such as SCFAs, during critical periods of development may represent a novel strategy for preventing or treating neurodevelopmental disorders characterized by abnormal synaptic pruning.

## Data Availability

No new data were created or analyzed in this study. Data sharing is not applicable to this article.

## Abbreviations

The following abbreviations are used in this manuscript:

BBB	Blood–brain barrier
CCL2	C-C motif chemokine ligand 2
CCL5	C-C motif chemokine ligand 5
CFC	Contextual fear conditioning
CTL	Control
CX3CR1	C-X3-C motif chemokine receptor 1
CX3CR4	C-X3-C motif chemokine receptor 4
dLGN	Dorsal lateral geniculate nucleus
E	Embryonic day
ELA	Early-life adversity
F	Female(s)
GF	Germ-free
HPC	Hippocampus
IGF1	Insulin-like growth factor 1
IL-1β	Interleukin-1 beta
IL-6	Interleukin-6
LB	Limited bedding
M	Male(s)
MEGF10	Multiple EGF-like domains protein 10
MG	Microglia
mPFC	Medial prefrontal cortex
NGF	Nerve growth factor
P	Postnatal day
P2RY12	Purinergic receptor P2Y12
PFC	Prefrontal cortex
PSD95	Postsynaptic density protein 95 (DLG4)
rsfMRI	Resting-state functional magnetic resonance imaging
SCFAs	Short-chain fatty acids
TNFα	Tumor necrosis factor alpha
TREM2	Triggering receptor expressed on myeloid cells 2
